# Understanding preschool teachers’ perceptions and intervention strategies for challenging behaviors in young children

**DOI:** 10.3389/fpsyg.2026.1887046

**Published:** 2026-07-20

**Authors:** Zhen Xiong, Xiaoyue Shen, Haomei Liu, Anqing Jiang, Jiaoni Yao

**Affiliations:** 1 College of Child Development and Education, Zhejiang Normal University, Hangzhou, Zhejiang, China; 2 Laboratory of Child Culture and Brain-Mind Development, Zhejiang Normal University, Hangzhou, Zhejiang, China; 3 Shuowang Kindergarten, Wuxi, Jiangsu, China

**Keywords:** attributions, behavioral characteristics, educational strategies, naughty children, preschool teacher

## Abstract

**Objective:**

This study aims to explore preschool teachers’ perceptions of “naughty children,” including their behavioral characteristics, attributed causes, and the educational strategies employed, in order to provide practical guidance for early childhood education.

**Methods:**

A mixed-methods design was adopted. Initially, in-depth interviews were conducted with 200 early childhood educators to capture qualitative insights into behavioral characteristics, attributions, and intervention strategies. Based on these interviews, an item pool was generated and refined through expert review. An exploratory factor analysis (EFA) was first conducted to identify the underlying structure, followed by Confirmatory Factor Analysis (CFA) to validate the measurement model. A total of 200 teachers completed the final validated questionnaire. Qualitative data were analyzed using NVivo 12, while quantitative data were subjected to Confirmatory Factor Analysis (CFA) and Structural Equation Modeling (SEM) to examine the factor structure and interrelationships among behavioral characteristics, attributions, and educational strategies. Measurement invariance across teacher subgroups was also assessed.

**Results:**

Teachers predominantly perceived “naughty children” as displaying weak rule awareness, self-centeredness, and aggressive or destructive tendencies, while positive traits such as creativity and curiosity were less frequently observed. Family upbringing was identified as the most frequently reported attribution factor, although the proportion of responses indicating this factor remained relatively limited (10.5%), with environmental triggers and individual developmental characteristics contributing to a lesser extent. Educational strategies primarily involved on-site interventions and home-school collaboration. SEM results revealed that family-related attributions were significantly and positively associated with the reported use of both on-site interventions (β = 0.42, *p* < 0.001) and home-school collaboration (β = 0.31, *p* < 0.001). CFA confirmed a three-factor structure encompassing behavioral characteristics, attributions, and educational strategies, with acceptable model fit indices.

**Conclusion:**

Preschool teachers’ perceptions of “naughty children” are associated with common biases and a limited understanding of the multidimensional causes of challenging behavior. Effective management requires recognizing positive traits, adopting holistic and professional approaches, and strengthening teacher training on behavioral assessment and intervention strategies. Early identification and systematic support can enhance both children’s development and teachers’ professional competence.

## 1 Introduction

In recent years, the development of behavioral habits among preschool children has become a key educational focus. At the policy level, both the *Guidelines for the Assessment of Quality in Care and Education* (2022) and the upcoming *Preschool Education Law* (2025) emphasize that character development is crucial for improving preschool education quality. In practice, educators increasingly prioritize character cultivation. Real-life incidents–disruptive public behavior, imitation of dangerous acts, school conflicts–highlight challenges in character development and the risk of stigmatization. These challenges arise from family education pressures, insufficient home-school collaboration, and social environmental stimuli, reinforcing public anxiety and prejudice. Thus, there is an urgent need to re-examine the concept of “naughty children,” its causes, and corresponding educational strategies from a professional perspective.

With the rise of short-video platforms, the term “naughty children” has become a social label carrying negative moral connotations (Wu et al., 2025). Public assessments based on fragmented clips often ignore the contextual and developmental nature of children’s behavior, conflicting with scientific parenting and child development principles. Both China’s *Law on the Promotion of Family Education* and the *Preschool Education Law* mandate that teachers provide family education guidance to parents. As dual-role educators and family education advisors, preschool teachers’ understanding of “naughty children” directly affects the quality of both preschool and family education (Shi et al., 2025). Therefore, exploring teachers’ perceptions of the behavioral characteristics, causes, and intervention strategies for “naughty children” can help correct public misconceptions and foster a more scientific, developmental, and supportive view of children and education (O’Grady and Ostrosky, 2024).

The term “naughty child” originated in northern and northeastern Chinese colloquial speech, describing mischievous or disobedient children. Fueled by media, it has evolved into a stigmatizing label denoting “deliberate bullying, harming others, and intentionally causing trouble” (Guo, 2023). The age range for such labeling has shifted from 5–12 years to 0–12 years (Hu, 2021), reflecting a misalignment of adults’ normative expectations of minors (Rad et al., 2022). Lemert (1972) proposed that everyone is a “primary deviant” who occasionally exhibits minor violations, but these can be self-regulated. However, primary deviants face labeling or stigmatization. In the digital age, a covert “panopticon-like mechanism” exacerbates child stigmatization, solidifying “naughty child” into a “problem child” category. Existing research has deepened from impact assessment to mechanisms of stigmatization and intervention, but little attention has been paid to how preschool teachers–key educators–perceive and educate “naughty children.”

Setting aside labeling, from an educational perspective, “naughty children” fall under challenging, disruptive, or problem behavior. Challenging behavior is defined as any behavior that interferes with optimal learning or prosocial interactions (Price and Steed, 2016). It includes self-harm, aggression, severe destruction, and violent tantrums, causing distress to the child or others (Zan, 2013). Such behaviors are frequent and persistent, manifesting externally or internally, reflecting difficulties in emotional regulation, social interaction, and self-control (Aksoy, 2020). Alter et al. (2013) categorized challenging behaviors into nine types (e.g., off-task, verbal/physical aggression, non-compliance). Aksoy (2020) identified four themes: adjustment issues, poor self-control, lack of prosocial behavior, and deficient self-confidence. Although “naughty” behaviors may deviate from social norms, they are not fully equivalent to “behavioral problems” (Zhang, 2019). Challenging behaviors stress teachers, affecting their efficacy and well-being (Miao et al., 2018), and diminishing teacher-child interaction quality.

Regarding causes, research indicates a combination of child, family, school, and society, with family upbringing as the primary factor. Despite young children’s egocentricity, limited social-moral awareness, and poor self-control, the absence or incorrect guidance from parents is the main cause (Li and Zeng, 2016; Yan and Zhang, 2019; Ren, 2023). Intervention strategies mainly involve home-school collaboration and positive guidance. Vygotsky and Cole (1978) “parental scaffolding” theory highlights parents’ cognitive, emotional, and autonomy support. Positive guidance approaches are commonly embedded within broader positive behavior support frameworks that emphasize proactive classroom management and structured behavioral prevention strategies (Sugai and Horner, 2009). However, these strategies rely heavily on expert guidance, and preschool teachers have not received systematic training to address challenging behaviors (You, 2024).

Overall, existing research has outlined public perceptions and intervention frameworks but rarely focused on teachers’ perspectives. Building on qualitative interviews with 200 teachers, we developed a structured questionnaire (200 valid responses) and used Confirmatory Factor Analysis (CFA) to validate the factor structure of behavioral characteristics, attributions, and educational strategies. We further employed Structural Equation Modeling (SEM) to examine the hypothesized relationships among behavioral characteristics, attributions, and educational strategies, which were theoretically grounded in an integrated framework combining labeling theory and sociocultural theory.

This study integrates two complementary theoretical perspectives to construct a coherent explanatory framework. First, Lemert’s labeling theory provides the macro-level explanation for how social and media environments shape the construction of “naughty children” as a deviant label. In this view, children’s behaviors are not inherently problematic but are socially interpreted and reinforced through labeling processes, particularly in digital media contexts where fragmented behavioral displays are amplified and generalized. Second, Vygotsky’s sociocultural theory operates at the micro-level of educational practice, explaining how preschool teachers interpret and respond to these labeled behaviors within culturally mediated classroom environments. From this perspective, teacher interventions are not neutral reactions but socially situated “scaffolding” processes shaped by cultural norms, institutional expectations, and pedagogical experience.

**FIGURE 1:**
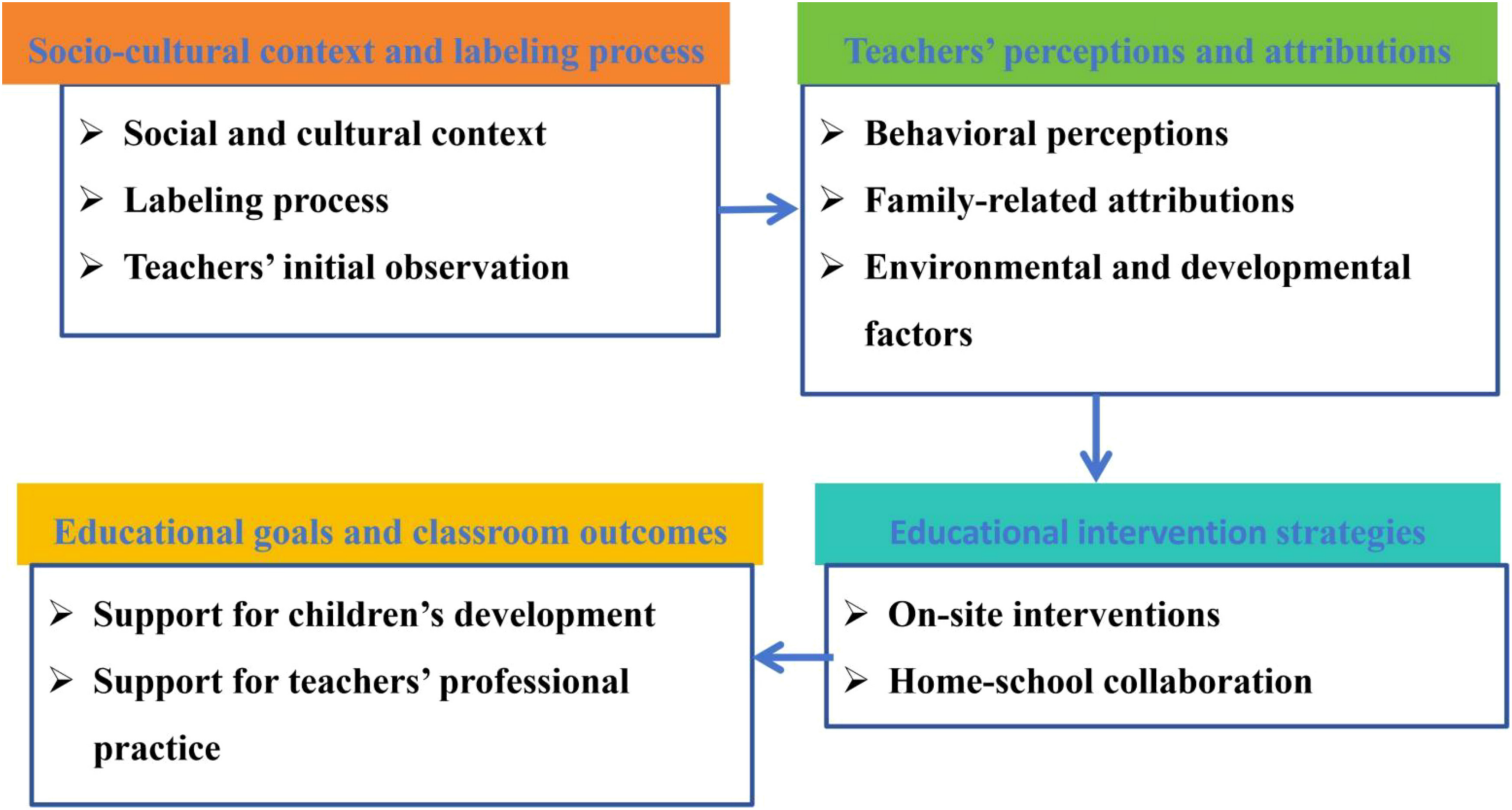
Conceptual model of associations among behavioral perceptions, attributions, and educational strategies based on labeling and sociocultural theory.

By integrating these two frameworks, this study proposes a conceptual model in which labeling processes, teachers’ cognitive attributions, and intervention strategies are examined as interrelated constructs within an SEM framework (Figure 1). SEM is therefore used not only as a statistical method but also as a structural representation of this theoretically grounded pathway. This study contributes to the literature by integrating labeling theory and sociocultural theory into a unified SEM framework to examine preschool teachers’ cognitive processes and behavioral responses in the context of challenging behaviors.

Based on the integrated theoretical framework combining labeling theory and sociocultural theory, this study addresses the following research questions: RQ1: What are preschool teachers’ perceptions of the behavioral characteristics of children they label as “naughty”? RQ2: What attributions do preschool teachers hold regarding the causes of these behaviors? RQ3: What educational strategies do preschool teachers employ in response to these behaviors? RQ4: How are teachers’ behavioral perceptions and causal attributions associated with their selection of intervention strategies?

RQ1–RQ3 are addressed through qualitative interviews and descriptive quantitative analysis, while RQ4 is examined through Structural Equation Modeling (SEM) that tests the hypothesized pathways from behavioral perceptions → attributions → intervention strategies.

## 2 Materials and methods

### 2.1 Design

This study adopted an exploratory sequential mixed-methods design, in which qualitative findings were systematically transformed into quantitative measures through a structured multi-step integration process (qualitative coding → item generation → expert validation → exploratory factor analysis → Confirmatory Factor Analysis). In the first qualitative phase, semi-structured interviews with 200 preschool teachers were conducted to generate initial themes regarding behavioral characteristics, attributions, and intervention strategies. In the second quantitative phase, these qualitative findings were transformed into measurable items to construct a structured questionnaire. The measurement development followed a rigorous psychometric process, including item generation, expert content validation, EFA for scale reduction and structure identification, and CFA for model validation.

In this study, the term “naughty children” is used as a culturally constructed label rather than a diagnostic category, and is consistently presented in quotation marks or as “children labeled as ‘naughty”’ to avoid stigmatizing terminology.

### 2.2 Participants and sampling

To ensure methodological clarity, the samples used in this study were independent across different research phases. A total of 200 preschool teachers participated in the qualitative interview phase. Within this sample, 33 participants were selected as a representative subsample for demographic description in Table 1.

**TABLE 1:** Demographic information of interview participants.

Number	Gender	Teaching tenure	Educational background	Kindergarten grade	Kindergarten type
1	Female	28	Bachelor degree	First-class	Public-run
2	Female	5	Bachelor degree	First-class	Public-run
3	Female	13	Bachelor degree	First-class	Public-run
4	Female	4	Bachelor degree	First-class	Public-run
5	Female	13	Bachelor degree	First-class	Public-run
6	Female	21	Bachelor degree	First-class	Public-run
7	Male	5	Bachelor degree	First-class	Public-run
8	Female	4	Bachelor degree	First-class	Public-run
9	Female	13	Bachelor degree	First-class	Public-run
10	Female	1	Bachelor degree	First-class	Public-run
11	Female	15	Master degree	First-class	Public-run
12	Female	5	Bachelor degree	First-class	Public-run
13	Female	6	Bachelor degree	First-class	Public-run
14	Female	1	Bachelor degree	First-class	Public-run
15	Female	9	Junior college	First-class	Public-run
16	Female	18	Bachelor degree	First-class	Private-run
17	Female	2	Bachelor degree	Third-class	Private-run
18	Female	11	Junior college	Third-class	Public-run
19	Female	7	Bachelor degree	First-class	Private-run
20	Female	3	Bachelor degree	Third-class	Private-run
21	Female	2	Bachelor degree	Third-class	Public-run
22	Female	4	Bachelor degree	First-class	Private-run
23	Female	6	Junior college	Third-class	Public-run
24	Female	3	Bachelor degree	First-class	Public-run
25	Female	6	Bachelor degree	Second-class	Public-run
26	Female	2	Bachelor degree	First-class	Public-run
27	Female	3	Bachelor degree	First-class	Public-run
28	Female	35	Bachelor degree	First-class	Public-run
29	Female	17	Bachelor degree	First-class	Public-run
30	Female	2	Junior college	First-class	Public-run
31	Female	15	Junior college	First-class	Public-run
32	Female	3	Bachelor degree	Second-class	Private-run
33	Female	15	Junior college	Third-class	Private-run

The pilot test (*n* = 33) involved a separate group of teachers who did not participate in either the qualitative interviews or the final survey. An independent sample of 120 teachers was used for EFA, while a separate sample of 200 teachers completed the final questionnaire for CFA and SEM. No overlap existed between the EFA and CFA/SEM samples, ensuring full independence across analytical stages.

The sample consisted of 186 female teachers (93.0%) and 14 male teachers (7.0%). Regarding teaching experience, 68 teachers (34.0%) had 5 or fewer years of experience, 52 teachers (26.0%) had 6–10 years, 46 teachers (23.0%) had 11–20 years, and 34 teachers (17.0%) had more than 20 years of experience. In terms of educational background, 38 teachers (19.0%) held a college diploma, 154 teachers (77.0%) held a bachelor’s degree, and 8 teachers (4.0%) held a postgraduate degree. Regarding kindergarten type, 142 teachers (71.0%) worked in public kindergartens and 58 teachers (29.0%) in private kindergartens. Kindergarten rankings included 108 teachers (54.0%) from first-class kindergartens, 52 teachers (26.0%) from second-class, and 40 teachers (20.0%) from third-class institutions. All participants were recruited from kindergartens in Zhejiang Province, China.

In this study, the term “naughty children” is treated as a culturally embedded social label in the Chinese context rather than a clinical diagnostic category. It differs fundamentally from internationally defined “challenging behavior,” which refers to observable behaviors that interfere with learning and social interaction without cultural or moral attribution. To avoid conceptual conflation, this study explicitly excluded children with clinically diagnosed neurodevelopmental disorders (e.g., ADHD, autism spectrum disorder) based on school medical records, parent-reported diagnostic documentation, or confirmed records in the kindergarten health management system, and focused only on non-pathological behavioral deviations as perceived by preschool teachers. This distinction was implemented by trained kindergarten administrative staff in collaboration with class teachers, ensuring that inclusion decisions were not based on teachers’ subjective judgment alone.

Specifically, prior to recruitment, kindergarten records were screened by administrative staff, followed by cross-checking with parents to confirm whether any formal clinical diagnosis had been made. Only children without documented diagnoses were included in the study.

### 2.3 Measures

Unstructured interviews captured teachers’ perceptions of “naughty children,” focusing on behavioral characteristics, attributions, and educational strategies. Behavioral characteristics included primarily observable externalizing behaviors such as rule-breaking, aggression, and self-control deficits. Internalizing behaviors (e.g., anxiety, withdrawal, and social avoidance) were not included as a separate measured dimension in the questionnaire but were discussed as a theoretical gap identified from qualitative coding. All interviews were audio-recorded, transcribed verbatim, and imported into NVivo 12 for analysis. A thematic analysis approach was employed, including open coding, axial coding, and selective coding. Two researchers independently coded the transcripts, and discrepancies were resolved through discussion to ensure analytical consistency. A coding tree was developed to consolidate recurring themes into higher-order categories, which subsequently informed the generation of questionnaire items. The classification of behavioral characteristics drew upon frameworks proposed by Powell et al. (2007) and Alter et al. (2013).

The structured questionnaire was developed from qualitative interview findings. Initially, an item pool was generated and reviewed by a panel of early childhood education experts for content validity. Subsequently, EFA was conducted to examine the underlying factor structure of the measurement model and to refine the item pool by removing low-loading and cross-loading items. Principal axis factoring with oblique rotation was used given the expected correlation among latent constructs. The refined scale was then subjected to CFA to validate the factor structure identified in the EFA stage and to test construct validity prior to SEM.

Based on the qualitative coding results, an initial item pool was generated to reflect teachers’ perceived behavioral characteristics, attributional explanations, and educational strategies. Redundant and ambiguous items were removed through iterative discussion among the research team. The refined item pool was then reviewed by a panel of early childhood education experts to ensure content validity, clarity, and cultural appropriateness. This process ensured that the quantitative instrument was directly grounded in qualitative findings rather than being independently developed. All analyses were conducted using AMOS 24 and SPSS 26.

#### 2.3.1 Item generation and scale development

Based on the qualitative coding results from the interviews, an initial item pool of 48 items was generated to reflect teachers’ perceived behavioral characteristics (18 items), attributional explanations (15 items), and educational strategies (15 items). Redundant and ambiguous items were removed through iterative discussion among the research team, resulting in a refined pool of 32 items.

#### 2.3.2 Expert content validation

The 32-item pool was then reviewed by a panel of five early childhood education experts (three university professors specializing in child development and two senior kindergarten directors with over 15 years of teaching experience). Experts were asked to evaluate each item for relevance, clarity, and cultural appropriateness on a 4-point content validity scale (1 = not relevant, 4 = highly relevant). The Item-level Content Validity Index (I-CVI) was calculated, and items with I-CVI below 0.78 were revised or removed. The Scale-level Content Validity Index (S-CVI/Ave) was 0.91, indicating excellent content validity.

#### 2.3.3 Pilot testing

A pilot test was conducted with 33 preschool teachers. Based on pilot feedback, four items were reworded for clarity and two items were removed due to low comprehension, resulting in a final 26-item questionnaire. The pilot data were not included in the final analysis.

#### 2.3.4 Exploratory factor analysis (EFA)

Exploratory factor analysis was conducted on the 26-item scale using data from an independent sample of 120 teachers. Principal axis factoring with oblique rotation (Promax) was used given the expected correlation among latent constructs. Items with factor loadings below 0.40 or cross-loadings above 0.32 were removed. The EFA yielded a three-factor structure corresponding to Behavioral Characteristics (8 items), Attributions (7 items), and Educational Strategies (6 items), explaining 58.3% of the total variance. The refined 21-item scale was then administered to the full sample of 200 teachers for CFA and SEM analyses.

### 2.4 Data collection

Interviews were audio-recorded with informed consent and conducted using a semi-structured interview protocol developed based on prior literature and expert consultation. The interview guide included questions on behavioral characteristics, perceived causes, and educational strategies related to “naughty children.” The full interview protocol is provided in Supplementary Appendix A. The transcriptions were organized chronologically, and thematic analysis was conducted to extract core concepts. The structured questionnaire was administered to a larger sample of 200 teachers to obtain quantitative data for validation of the factor structure and structural relationships identified in the qualitative analysis.

### 2.5 Data analysis

Data analysis followed a two-step measurement validation procedure consistent with exploratory sequential design principles. First, qualitative data were analyzed using NVivo 12 to generate themes that informed item development. Second, EFA was used to identify latent constructs and refine the measurement scale. Third, CFA was conducted to validate the factor structure prior to SEM. This sequential analytic strategy ensured alignment between qualitative findings and quantitative measurement.

Given the imbalance in subgroup sample sizes, particularly the limited representation of male teachers, subgroup comparisons were conducted at a descriptive level only. The distribution of teaching experience and kindergarten type suggested similar response patterns across groups; however, inferential multi-group Structural Equation Modeling was not performed due to insufficient statistical power. Therefore, the structural model was estimated using the full sample to ensure robustness and stability of parameter estimates.

## 3 Results

### 3.1 Questionnaire development and factor structure

Table 1 shows 33 of 200 interviews, key themes were extracted and transformed into questionnaire items rated on a 5-point Likert scale. Core latent factors were defined as follows: (1) Behavioral Characteristics (e.g., weak sense of rules, self-centeredness, aggressive/destructive tendencies), (2) Attributions (family upbringing, environmental triggers), and (3) Educational Strategies (on-site interventions, home-school collaboration).

### 3.2 Descriptive statistics

The survey yielded 200 valid responses. Descriptive analyses indicated that the most frequent negative behavioral characteristics were weak rule awareness (*M* = 4.2, SD = 0.7), self-centeredness (*M* = 3.9, SD = 0.8), and poor self-control (*M* = 4.0, SD = 0.7), while positive traits such as curiosity and creativity were less frequently observed (*M* = 3.5, SD = 0.8). Items in Table 2 were rated on a 5-point Likert scale. To illustrate the thematic categories derived from the qualitative interviews, representative teacher quotes are presented below. For behavioral characteristics, one teacher remarked: “This child never follows the rules, always runs around and interrupts others–he just doesn’t listen” (T12). Another teacher noted: “She is very self-centered, always grabbing toys and refusing to share” (T7). Regarding attributions, a teacher stated: “I think most of the problems come from the family–parents spoil the child too much and give no boundaries” (T23). In contrast, a few teachers acknowledged positive aspects: “He is actually very curious and asks lots of questions, but sometimes his energy gets out of hand” (T5). For intervention strategies, a teacher described: “When a child acts out, I usually stop the activity and talk to him immediately, then later I call the parents to discuss consistency” (T18). Another teacher used encouragement: “I try to praise him when he does something good, so he knows what is expected” (T31).

**TABLE 2:** Questionnaire items and core latent factors.

Element	Key concepts	Questionnaire items/description
Behavioral characteristics	Non-compliance	Lack of awareness of rules, no sense of rules, failure to follow rules, breaching class rules, flouting rules
	Lack of self-control	Aggressive behavior, provocation, teasing, mischief, vandalism, frequent conflicts, snatching things, causing disruption
	Lack of prosocial behavior	Disregarding others’ feelings, self-centered, self-absorbed, willful, refusing to follow instructions
Positive characteristics	Lively and spirited	Imaginative, inquisitive, clever, original, quick-witted, cheerful, full of energy, curious, keen to explore
Attributions	Family upbringing	Parental influence, overbearing parents, excessive pampering, lack of attention, indulgence, intergenerational conflicts
	Environmental triggers	School education, peer influence, living environment
Educational strategies	On-site interventions	De-escalation, immediate intervention, behavioral analysis, positive reinforcement, group education
	Home-school collaboration	Communication with parents, home-school partnership, educational consistency

Table 3 integrates attributional factors and educational strategies based on the quantitative survey of 200 valid responses. All frequencies and percentages were recalculated using the full sample size (*N* = 200) to ensure consistency and statistical accuracy.

**TABLE 3:** Causes and educational strategies for “naughty children” based on teacher survey data.

Causes of “naughty children”-attribution category	Subcategory	Frequency (*n*)	Percentage (%)
Family upbringing	General family education	21	10.50%
	Overprotection and indulgence	10	5.00%
	Lack of emotional support	5	2.50%
	Negative parental role modeling	1	0.50%
Individual development	Innate temperament	8	4.00%
Environmental triggers	Peer misconduct	2	1.00%
Educational strategies-strategy type	Sub-strategy	Frequency (*n*)	Percentage (%)
On-site intervention	Behavioral analysis	20	10.00%
	Behavioral intervention/cold shoulder	12	6.00%
	Positive reinforcement	4	2.00%
	Group education	2	1.00%
Home-school collaboration	Communication with parents	10	5.00%
	Parenting guidance	2	1.00%
	Emotional support	2	1.00%

Frequencies represent the number of mentions coded at the response level rather than mutually exclusive teacher-level selections. Each participant could report multiple attributional categories and intervention strategies; therefore, percentages are calculated based on total valid responses (*N* = 200), and cumulative percentages may exceed 100%. Coding was performed at the response unit level, and two independent coders conducted classification with discrepancies resolved through consensus.

### 3.3 Confirmatory Factor Analysis (CFA)

**FIGURE 2:**
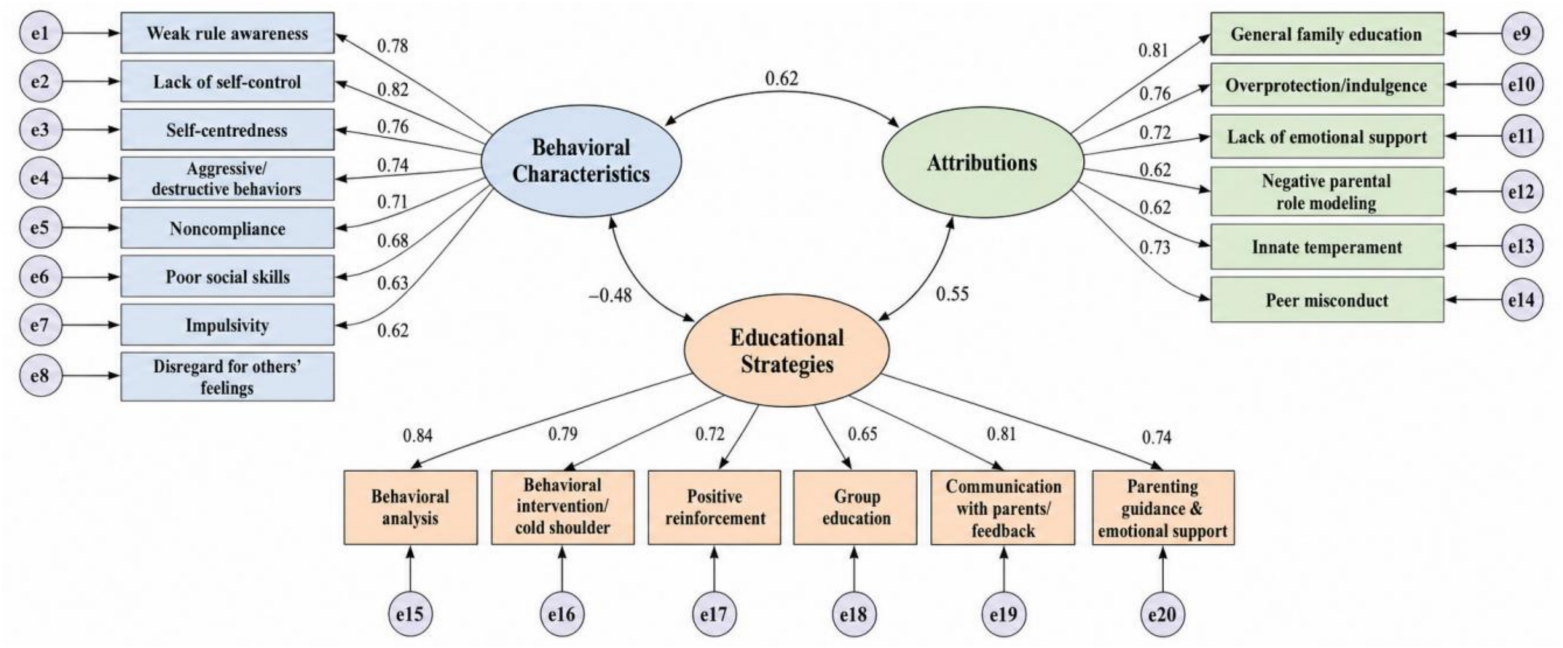
Confirmatory Factor Analysis (CFA) path diagram of the three-factor model.

Confirmatory Factor Analysis was conducted to validate the latent factor structure of the questionnaire. The three-factor model–Behavioral Characteristics, Attributions, and Educational Strategies–demonstrated good fit (CFI = 0.95, TLI = 0.94, RMSEA = 0.05, SRMR = 0.04). As shown in Figure 2, each latent factor is represented by an ellipse with its associated observed variables, and standardized factor loadings ranged from 0.62 to 0.84, indicating that all items significantly contributed to their respective latent constructs. Internalizing behaviors such as anxiety and withdrawal were not directly measured in the quantitative scale and did not emerge as an independent factor in the CFA structure; however, qualitative coding suggested that such behaviors were rarely mentioned by teachers compared with externalizing behaviors.

### 3.4 Structural Equation Modeling (SEM)

Structural Equation Modeling was applied to explore the relationships among the latent variables. The structural model demonstrated good fit to the data: χ^2^(186) = 312.45, *p* < 0.001; CFI = 0.94; TLI = 0.93; RMSEA = 0.058 (90% CI [0.048, 0.068]); SRMR = 0.045. All fit indices met or exceeded recommended thresholds (CFI ≥ 0.90, TLI ≥ 0.90, RMSEA ≤ 0.08, SRMR ≤ 0.08), indicating acceptable model fit.

**FIGURE 3:**
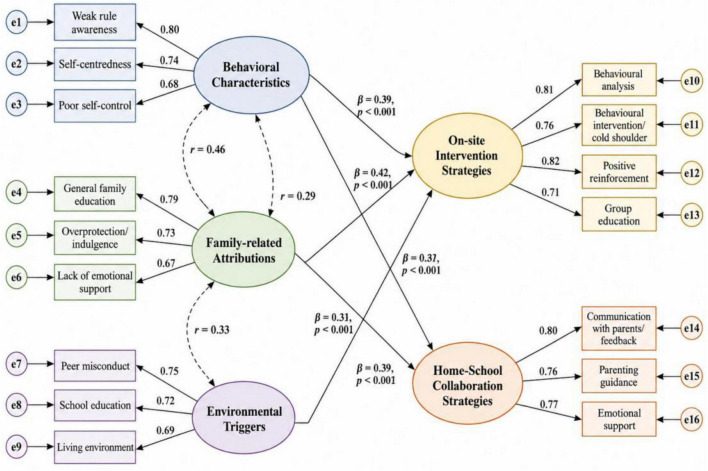
Structural Equation Model of behavioral characteristics, attributions, and educational strategies.

As shown in Figure 3, family-related attributions were positively associated with on-site intervention strategies (β = 0.42, *p* < 0.001) and home-school collaboration strategies (β = 0.31, *p* < 0.001). Environmental triggers were also significantly associated with on-site interventions (β = 0.18, *p* = 0.03). Behavioral characteristics, particularly weak rule awareness and self-centeredness, were significantly associated with both educational strategies (β = 0.39, *p* < 0.001). The arrows in Figure 3 indicates the associational relationships estimated within the SEM framework, with standardized coefficients labeled on each connection. These results indicate a stable structural model with statistically significant associations among constructs, without implying causal directionality.

## 4 Discussion

This mixed-methods study examined preschool teachers’ perceptions, attributions, and intervention strategies regarding children labeled as “naughty” in Chinese early childhood settings. The findings reveal a coherent interpretive framework: teachers predominantly characterize such children through externalizing behavioral indicators, attribute these behaviors primarily to family factors, and respond with reactive rather than preventive strategies. This pattern, empirically supported by the SEM results, indicates an associational pattern among teachers’ behavioral perceptions, attributions, and reported intervention strategies, rather than implying directional or causal effects. Below, we discuss each major finding in turn, situate them within the broader literature, and draw out theoretical and practical implications.

### 4.1 Teachers’ predominant focus on externalizing behaviors and underrecognition of internalizing difficulties

A central finding of this study is that preschool teachers predominantly perceive “naughty children” through the lens of externally observable behaviors–particularly weak rule awareness, self-centeredness, and aggressive or destructive tendencies–while internalizing behaviors such as anxiety, withdrawal, and social isolation remain largely overlooked. This pattern was consistent across both qualitative interviews and quantitative survey data, where weak rule awareness emerged as the most salient behavioral indicator (*M* = 4.2, SD = 0.7). This finding aligns with previous research in preschool settings showing that teachers tend to notice and respond to behaviors that disrupt classroom order, while more covert difficulties receive less attention (Dal and Akan, 2018; Güder et al., 2018).

The tendency to prioritize externalizing behaviors is understandable from a classroom management perspective: rule-breaking and aggression pose immediate safety risks and disrupt instructional continuity, whereas internalizing behaviors are quieter and less likely to trigger urgent responses. However, this selective attention carries significant costs. Internalizing difficulties in early childhood–if unaddressed–can predict later anxiety disorders, depressive symptoms, and social withdrawal (Whitcomb, 2013; Aksoy, 2020). Teachers who fail to recognize these covert indicators may miss critical opportunities for early support and intervention. This finding echoes broader concerns in the child development literature about the “hidden” nature of internalizing problems and the tendency for such difficulties to be normalized or overlooked in educational settings (Uyanık et al., 2008).

Furthermore, our results suggest that teachers’ descriptions of “naughty” behavior tend toward trait-based characterizations–using labels such as “self-centered” or “lacking self-control”–rather than situationally grounded descriptions of specific behaviors. This distinction is theoretically important. Behavioral descriptions invite reflection on environmental contingencies, activity design, and teacher–child interaction patterns, while trait attributions are more likely to evoke halo effects, self-fulfilling prophecies, and stigmatization (Qian and Qi, 2011; Zhang et al., 2020). On the one hand, child stigmatization reduces social status and self-perception (Qian and Qi, 2011), triggers shame and inferiority, lowers self-efficacy, and leads to withdrawn behavior (Zhang et al., 2020; Sun, 2025). On the other hand, it can manifest as public, self, associative, and vicarious stigma (Sun, 2025). Public spaces increasingly exhibit pre-emptive stigmatization and preventive discipline–for example, high-speed train crew reminders to “keep children under control” (Upstream News, 2023). Cossaro further notes that children are often viewed as troublemakers in the adult world (Zheng and Yang, 2024). Thus, teachers should avoid publicly using labeling terms such as “naughty children” or “developmentally delayed.” Labeling a child as “naughty” in global terms may obscure the contextual and developmental nature of challenging behavior, reducing teachers’ willingness to examine how classroom environments or instructional approaches might be modified to better support the child.

Although teachers in our sample predominantly emphasized negative traits, a small subset offered positive interpretations from a developmental perspective, noting qualities such as curiosity, creativity, and spiritedness alongside behavioral challenges. This minority perspective is consistent with developmental theories that frame challenging behavior as an expression of children’s ongoing exploration and adaptation to their environments (Zuo et al., 2012). However, the relatively low endorsement of positive traits (*M* = 3.5, SD = 0.8) suggests that such developmental framings remain marginal in teachers’ everyday conceptualizations.

### 4.2 The dominance of family attribution and its consequences

The SEM results revealed that, among the attributional factors examined, family-related attributions were most strongly associated with teachers’ reported intervention choices, showing significant associations with both on-site interventions (β = 0.42, *p* < 0.001) and home–school collaboration (β = 0.31, *p* < 0.001). This finding is consistent with the qualitative interviews, in which most teachers emphasized parental influence–including overprotection, indulgence, and inconsistent discipline–as the primary cause of challenging behavior. While family factors undoubtedly play a significant role in children’s behavioral development, the overwhelming dominance of this single attributional pattern raises important concerns.

First, a singular focus on family factors may lead teachers to overlook other contributing dimensions, including child-specific factors (e.g., temperament, developmental stage, self-regulation capacity), environmental triggers (e.g., peer dynamics, classroom structure, transition-related stress), and broader societal influences. Challenging behavior in young children is almost invariably multiply determined, emerging from the dynamic interplay of child characteristics, family context, and environmental demands (Powell et al., 2007; Uyanık et al., 2008). Teachers who rely on a simplified attributional model may fail to identify modifiable classroom factors that could be adjusted to prevent or reduce challenging behavior. This concern is amplified by evidence suggesting that comprehensive behavioral assessment should integrate multiple sources of information and consider the full ecological context in which behavior occurs (Zan, 2013).

Second, strong family attributions may subtly shift responsibility away from the educational setting, positioning teachers as reactors to problems originating elsewhere rather than as agents capable of shaping behavioral outcomes through classroom practices. This orientation may reduce teachers’ sense of efficacy in managing challenging behavior and limit their engagement in proactive, preventive strategies. Existing research has shown that teachers’ attributions for student behavior significantly influence their expectations and instructional decisions (Clayback and Williford, 2023); Our findings extend this insight to the early childhood context, indicating that attributional patterns are strongly associated with the types of strategies teachers select.

Third, the tendency to attribute challenging behavior primarily to family factors may inadvertently communicate blame or judgment to parents, potentially undermining the collaborative relationships that are essential for effective home–school partnership. Although teachers in our sample reported using home–school collaboration as a key strategy, the attributional framework underlying such collaboration may shape whether parent–teacher communication is experienced as supportive or accusatory. Future research should examine how attributional patterns influence the quality and outcomes of home–school partnerships. This finding suggests that family environment is a relatively more salient explanatory framework used by teachers, although it does not dominate the attribution distribution in absolute terms. It should be noted that although family-related attributions were the most frequently selected category, the overall distribution indicates a dispersed attribution pattern rather than a single dominant causal explanation. Finally, while this study integrates labeling theory and sociocultural theory within an SEM framework, the theoretical integration remains at a preliminary stage. The explanatory power of this combined framework–particularly regarding the mediating mechanisms linking attributions to strategy selection–requires further conceptual refinement. Moreover, although the qualitative interviews generated rich thematic content, their interpretive depth is constrained by the semi-structured format and the primary goal of instrument development. Future research could employ ethnographic or longitudinal qualitative designs to achieve deeper contextual understanding and to examine the dynamic processes underlying teachers’ perceptions and interventions. These limitations are inherent to the exploratory sequential design, which prioritized measurement development over comprehensive theoretical elaboration.

### 4.3 Reactive versus proactive strategies: implications for professional development

Teachers in this study reported using two primary categories of strategies: on-site interventions (e.g., behavioral analysis, immediate redirection, and in some cases, planned ignoring) and home–school collaboration (e.g., parent communication, parenting guidance). While both strategy types reflect reasonable responses to challenging behavior, they are predominantly reactive in nature–focused on managing or stopping misbehavior after it has occurred–rather than proactive approaches designed to prevent challenging behavior through environmental modification, skill-building, or positive behavior support.

This reactive orientation is consistent with broader patterns observed in early childhood education. Aksoy (2020) found that preschool teachers often employ consequence-based strategies such as verbal warnings, time-out, and removal of privileges, while less frequently using antecedent-based strategies that address the conditions underlying challenging behavior. Similarly, Culp et al. (2001) demonstrated that teachers’ responses to children’s problem behavior can either ameliorate or exacerbate difficulties, depending on the quality and timing of the intervention. The predominance of reactive strategies in our sample suggests a need for professional development that equips teachers with a broader repertoire of proactive, preventive approaches.

Notably, a small number of teachers in our qualitative interviews described strategies consistent with encouragement-based approaches, emphasizing positive reinforcement and relational support rather than punishment or ignoring. Such practices align with Dreikurs and Stolz’ (1991) emphasis on encouragement as a means of fostering children’s sense of belonging and capability. However, these examples were exceptional rather than typical, suggesting that such approaches remain underutilized in everyday practice.

The CFA results confirmed that behavioral characteristics, attributions, and educational strategies form distinct but related latent constructs, with the three-factor model demonstrating acceptable fit (CFI = 0.95, TLI = 0.94, RMSEA = 0.05, SRMR = 0.04). This measurement structure provides empirical support for the theoretical distinction among these domains and suggests that interventions targeting one domain (e.g., teachers’ attributional patterns) may indirectly influence others (e.g., strategy selection). The SEM results further clarified these relationships, suggesting that behavioral perceptions and family attributions are both associated with teachers’ strategic choices, with attribution showing the larger standardized coefficient.

The reactive orientation also has implications for how challenging behavior is conceptualized within the broader framework of positive behavior support. Although this study did not directly measure implementation of positive behavior support frameworks, the finding that teachers predominantly use reactive strategies suggests alignment with the need for proactive classroom management approaches that emphasize environmental design, explicit behavioral expectations, and systematic teaching of social-emotional skills (Sugai and Horner, 2009). Teachers in our sample appeared to have limited access to such frameworks, reinforcing calls for enhanced professional development in this area.

### 4.4 Theoretical integration: labeling, attribution, and the sociocultural context

The findings of this study can be conceptually integrated through the dual theoretical lens of labeling theory and sociocultural theory. From the perspective of labeling theory (Lemert, 1972), teachers’ use of the term “naughty” functions as a labeling act that may precede and enable stigmatization. When teachers describe children using global trait labels–“self-centered,” “lacking self-control,” or simply “naughty”–they implicitly categorize children in ways that may shape subsequent perceptions, reduce tolerance for normative behavioral variability, and create self-fulfilling prophecies. Categorization is essential for thinking but inevitably leads to preconceptions (Allport, 1954). Practitioners must avoid assumptions without sufficient evidence. This labeling process is further amplified by the sociocultural context in which teachers operate, including the influence of digital media and public discourse that increasingly frame children’s challenging behavior through moralizing and stigmatizing language (Guo, 2023; Hu, 2021). The term “naughty child” originated in northern and northeastern Chinese colloquial speech and, fueled by media, has evolved into a stigmatizing label denoting “deliberate bullying, harming others, and intentionally causing trouble” (Guo, 2023). The age range for such labeling has shifted from 5–12 years to 0–12 years (Hu, 2021), reflecting a misalignment of adults’ normative expectations of minors (Rad et al., 2022).

From the perspective of sociocultural theory (Vygotsky and Cole, 1978), teachers’ interpretations and responses to challenging behavior are not purely individual cognitive processes but are shaped by culturally mediated tools, including professional training, institutional expectations, and prevailing discourses about child development and parenting. Teachers in our sample operated within a cultural context where family upbringing is widely viewed as the primary determinant of children’s behavior, a belief that may be reinforced by policy frameworks emphasizing home–school collaboration and parental responsibility. This cultural framing may explain why family-related attributions were so strongly associated with intervention choices, even when alternative explanations (e.g., environmental triggers, developmental factors) were also acknowledged.

Together, these theoretical perspectives suggest that teachers’ perceptions and responses to “naughty” behavior are shaped by both macro-level sociocultural processes (labeling, stigmatization, media influence) and micro-level interpretive frameworks (attribution, scaffolding, professional reasoning). Future interventions aimed at improving teachers’ responses to challenging behavior should address both levels: reducing stigmatizing labels and promoting more nuanced, behaviorally grounded descriptions at the discursive level, while simultaneously enhancing teachers’ capacity for multidimensional behavioral assessment and proactive intervention at the pedagogical level.

### 4.5 Practical implications and teacher training

The findings of this study carry several practical implications for early childhood education policy and practice. First, there is an urgent need to incorporate child assessment and positive behavior support into preservice and in-service teacher training. Teachers in our sample demonstrated limited familiarity with systematic behavioral observation and assessment frameworks, relying instead on intuitive, category-based judgments. Training programs should equip teachers with practical tools for identifying both externalizing and internalizing behavioral indicators, using multiple sources of information (e.g., observation, parent report, child self-report where feasible), and differentiating between transient developmental behaviors and patterns that warrant more intensive support.

Second, professional development should explicitly address attributional patterns and their consequences. Teachers who attribute challenging behavior primarily to family factors may benefit from exposure to ecological and developmental frameworks that highlight the interplay of multiple influences, including classroom environment, peer relationships, and child-specific characteristics. Such training could help teachers move beyond single-source attributions and develop more differentiated, context-sensitive understandings of children’s behavior.

Third, efforts to reduce stigmatization of children labeled as “naughty” should be prioritized at both the classroom and policy levels. Teachers should be encouraged to use behaviorally descriptive language–specifying what the child did, under what conditions, with what consequences–rather than global trait labels that invite negative attributions. This shift in language is not merely semantic; it has direct implications for how teachers perceive children, design interventions, and communicate with families. The principles of the Mosaic Approach (Clark and Moss, 2011), which emphasizes understanding children through multiple perspectives and methods, offer a useful framework for this endeavor, though its implementation requires institutional support and ongoing reflective practice. The Guidelines for the Evaluation of Early Years Education and Care identify scientific evaluation as a core principle. Teachers should encourage diverse stakeholders to use multiple methods to understand children and reconstruct the meaning of their work.

Fourth, the reactive orientation observed in our sample suggests a need for systemic supports that enable teachers to implement preventive, proactive strategies. This includes reducing class sizes, ensuring adequate staffing ratios, providing access to early childhood mental health consultation, and creating institutional cultures that value positive behavior support over punitive or exclusionary practices. Teachers cannot be expected to implement proactive strategies without the structural conditions that make such approaches feasible.

Fifth, with the advancement of inclusive early childhood education and the rising proportion of children exhibiting challenging behaviors, there is an urgent need to incorporate child assessment courses into teacher training. Although system-level factors such as class size, staffing ratios, and access to mental health consultation may influence teachers’ ability to manage challenging behaviors, these variables were not directly measured in the present study and therefore should be interpreted as contextual considerations rather than empirically tested conclusions. These structural factors have been highlighted in previous literature as important supports for early childhood education quality; however, they remain outside the scope of the current dataset. Existing research indicates that children’s problem behaviors are a primary cause of negative emotions among early years teachers (Li, 2017; Sutton, 2000) and affect teacher-child interaction quality. Early prevention, scientific identification, and appropriate intervention should be shared priorities. Given the limited training reported by teachers in managing challenging behaviors, the findings suggest a need for strengthening practical, rather than purely theoretical, pedagogical support. Future training should prioritize helping teachers better interpret behavioral signals and develop context-sensitive intervention strategies grounded in classroom realities.

### 4.6 Limitations and future directions

Several limitations should be acknowledged when interpreting the findings of this study. First, the generalizability of the results may be limited by the sample composition. The qualitative sample included only one male teacher, and the quantitative data were primarily collected from kindergartens in Zhejiang Province. Although the sample size (*N* = 200) is acceptable for Structural Equation Modeling, broader regional and demographic diversity would strengthen external validity. Future studies should recruit more diverse samples across different provinces, kindergarten types, and teacher demographic characteristics.

Second, the cross-sectional design limits causal inference, even though SEM provides evidence of directional associations among behavioral perceptions, attributions, and intervention strategies. Longitudinal designs are needed to further validate these structural relationships and to examine how teachers’ perceptions and practices evolve over time, particularly in response to professional development or changes in classroom contexts.

Third, reliance on self-reported data may introduce social desirability bias, potentially leading to underreporting of negative perceptions or overreporting of positive intervention strategies. The absence of direct classroom observations or child outcome measures limits ecological validity. Future research should incorporate observational methods, child-level behavioral assessments, and multi-informant data (e.g., parent reports, peer sociometric data) to triangulate findings and strengthen robustness.

Fourth, although we measured teachers’ perceptions and attributions, we did not directly assess stigmatization processes or children’s experiences of being labeled. The discussion of stigma and labeling is therefore theoretical rather than empirical. Future studies should examine how children perceive and internalize teachers’ behavioral labels, and how such labeling processes affect children’s self-concept, peer relationships, and behavioral trajectories over time.

Fifth, although both exploratory factor analysis (EFA) and Confirmatory Factor Analysis (CFA) were conducted to ensure measurement validity, the relatively modest sample size may still affect the stability of factor solutions. In addition, measurement invariance across gender and other subgroups was not formally tested due to insufficient subgroup sizes, and therefore multi-group comparisons should be interpreted with caution.

## 5 Conclusion

Overall, the findings of this study suggest a consistent pattern from teachers’ behavioral perceptions to causal attributions and finally to intervention strategies. This structural pattern is statistically supported by the SEM results and conceptually grounded in an integrated framework combining labeling theory and sociocultural theory. The results suggest that teachers’ reported decision-making patterns are more closely aligned with attributional simplification than with structured behavioral classification systems, as reflected in the observed associations.

Teachers predominantly characterize “naughty children” by weak rule awareness, self-centeredness, and aggressive tendencies, with family upbringing perceived as the primary cause. Educational strategies are largely reactive (on-site interventions and home-school collaboration) rather than proactive. SEM results showed that family-related attributions were significantly associated with both on-site and home-school strategies, with family attributions showing the strongest relative association among the factors examined. Teachers tend to overlook internalized challenging behaviors and positive traits, and their singular focus on family factors limits holistic understanding and effective intervention.

To address these gaps, teacher training programmes must incorporate child assessment, positive behavior support, and strategies to reduce stigmatization. Early identification, scientific evaluation, and ecosystem-based interventions are essential. This study contributes empirical evidence from the under-researched teacher perspective, offering practical implications for improving early childhood education quality and fostering both children’s development and teachers’ professional competence.

## Data Availability

The raw data supporting the conclusions of this article will be made available by the authors, without undue reservation.
