# Supplementary material for: Understanding preschool teachers’ perceptions and intervention strategies for challenging behaviors in young children
Source: Front Psychol. 2026 Jul 20;17:1887046. doi: 10.3389/fpsyg.2026.1887046 (PMC13429590; doi:10.3389/fpsyg.2026.1887046)
Supplement: Supplementary file 1 [file Supplementary_file_1.docx]

Appendix A. Semi-Structured Interview Protocol

This interview protocol was developed to explore preschool teachers’ perceptions of “naughty children,” including behavioral characteristics, perceived causes, and educational intervention strategies. It was designed for an exploratory sequential mixed-methods study.

# 1. Introduction and Informed Consent

Before the interview, participants were informed about the purpose of the study, confidentiality, voluntary participation, and their right to withdraw at any time. Written informed consent was obtained from all participants.

# 2. Background Information

Please tell us about your teaching experience, including years of service, age group taught, and educational background.

# 3. Core Interview Questions

## 3.1 Understanding of “Naughty Children”

1. How do you define or understand the term “naughty children” in your daily teaching practice?

2. What types of behaviors do you associate with this label?

Probes: Can you give specific examples from your classroom experience?

## 3.2 Perceived Behavioral Characteristics

1. What behavioral characteristics do you most commonly observe in children you consider ‘naughty’?

2. Do you notice any positive characteristics in these children?

Probes: How do these behaviors affect classroom management and learning activities?

## 3.3 Attribution of Causes

1. In your opinion, what are the main causes of these behaviors?

2. To what extent do you think family, school, or individual factors contribute?

Probes: Can you explain a specific case that influenced your view?

## 3.4 Educational Strategies

1. What strategies do you usually use when dealing with such behaviors?

2. What strategies do you find most effective or ineffective?

Probes: How do you communicate with parents in these situations?

## 3.5 Teacher Reflection

1. How do these experiences influence your teaching beliefs or emotions?

2. What support or training would help you better handle such situations?

# 4. Closing Statement

Thank you for your participation. Your responses are highly valuable for understanding preschool teachers’ perceptions and improving early childhood education practices.
